# Evaluating the impact of statin therapy on chronic obstructive pulmonary disease outcomes: A retrospective cohort study from a tertiary care hospital in India

**DOI:** 10.1177/03000605261417046

**Published:** 2026-04-09

**Authors:** Harini Singara Chari, Ambika M Padukone, Rahul Magazine, P Kalyana Chakravarthy, Archisha Kalra, Bharti Chogtu

**Affiliations:** 1 Department of Respiratory Medicine, Kasturba Medical College, India; 2 Department of Pulmonary, Critical care and Sleep Medicine, AIIMS New Delhi, India; 3 Department of Respiratory Medicine, Kasturba Medical College, Manipal Academy of Higher Education, Manipal, India; 4 Department of Public Health Dentistry, 29228 Manipal College of Dental Sciences, Manipal Academy of Higher Education, Manipal, India; 5 Kasturba Medical College, Manipal Academy of Higher Education, Manipal, India; 6 Department of Pharmacology, Kasturba Medical College, Manipal Academy of Higher Education, Manipal, India

**Keywords:** Chronic obstructive pulmonary disease, statins, exacerbations, hospitalization, retrospective study, India

## Abstract

**Objective:**

Chronic obstructive pulmonary disease is a common condition marked by significant disability and frequent exacerbations. Statins, owing to their anti-inflammatory and immunomodulatory effects, may offer therapeutic benefits in chronic obstructive pulmonary disease management. This retrospective cohort study evaluated the impact of statin use on chronic obstructive pulmonary disease outcomes in a subset of Indian population.

**Methods:**

Patients diagnosed with chronic obstructive pulmonary disease from January 2015 to December 2019 were divided into two equal groups of 83 patients each: statin users and nonusers. Primary outcomes included the annual exacerbation rate and number of hospitalizations, while secondary outcomes assessed exacerbation severity, emergency room visits, and duration of hospital stay over 1 year.

**Results:**

Statin users showed a lower annual exacerbation rate (2.98 ± 1.9) compared to nonusers (3.51 ± 2.1), although this difference was not statistically significant (p = 0.058). Similarly, statin users had fewer hospitalizations (173 vs. 186; p = 0.493). However, the total duration of inpatient stays was significantly reduced in the statin group (1343 vs. 1518 days; p = 0.001). Outpatient and emergency visits were also lower among statin users, although these differences were not statistically significant.

**Conclusion:**

Statin use in patients with chronic obstructive pulmonary disease exhibited a trend toward improved outcomes, particularly in reducing hospital stay duration, suggesting potential benefits that warrant further investigation.

## Introduction

Chronic obstructive pulmonary disease (COPD) is a prevalent condition characterized by partially reversible airflow obstruction that progressively worsens over time. It is often triggered by exposure to external irritants and gases.^1^ As per the World Health Organization, “COPD is the third leading cause of death worldwide, causing 3.23 million deaths in 2019.”^2^ Smoking cessation, lung volume reduction surgery, and use of long-term oxygen therapy are the only treatment options that positively affect disease progression or mortality. Other available therapeutic interventions primarily provide symptomatic improvement.^3^

Chronic airway inflammation and remodeling, secondary to the influx of macrophages, neutrophils, and T lymphocytes, predominantly affecting the smaller airways, is very common in patients with COPD. Oxidative stress, imbalance in protease–antiprotease activity, and mitochondrial dysfunction contribute to increased cell apoptosis and subsequent emphysematous changes.^4^

In COPD, there is a need for newer therapies that can positively impact the inflammatory cascade. Studies have shown that apart from their plasma cholesterol-lowering properties, statins possess anti-inflammatory properties capable of significantly reducing interleukin-6 (IL-6) and C-reactive protein (CRP) levels.^5^ Hence, statins may potentially have a positive impact on COPD-related outcomes.

The primary objective of this study is to analyze whether statin use is associated with improved COPD-related outcomes, such as the frequency and severity of exacerbations and duration of hospital stay. Additionally, the study explores whether comorbidities such as ischemic heart disease and diabetes mellitus influence these outcomes.

## Methodology

### Study design and research setting

This was a retrospective cohort analysis undertaken at a tertiary care teaching hospital in Karnataka. The data were collected from the hospital’s medical record section. All consecutive patients diagnosed with COPD between January 2015 and December 2019 were identified using the International Classification of Diseases, Tenth Revision codes. They were grouped into statin users and nonstatin users, with 83 patients in each group. Follow-up data for 1 year were extracted from the records for all participants in the sample. Ethical clearance was obtained from the Institutional Ethics Committee (Letter number 638/2020 dated 23 February 2021). The Institutional Ethics Committee granted a waiver of informed consent in accordance with the norms of ICMR-2017 ethical guidelines for health research in India, as the study involved only the analysis of anonymized patient data collected during routine clinical care. This study was conducted in accordance with the ethical principles of the Declaration of Helsinki (1975, as revised in 2024).

### Study population and data collection

The diagnosis of COPD was established using the Global Initiative for Chronic Obstructive Lung Disease (GOLD) guidelines,^6^ and all the patients enrolled in the study were monitored over a 1-year period. Patients with follow-up durations of less than 1 year and those with other chronic pulmonary conditions, such as bronchial asthma or pulmonary fibrosis, were excluded. Additionally, patients with incomplete medical records, nonstatin lipid-lowering medication use, and persistent smoking were also excluded. Demographic and clinical characteristics were recorded from patient files, while data on investigations and radiological imaging were obtained from the hospital’s electronic database. The collected data were recorded in a predefined format. All patient identifiers were removed before the analysis. The dataset was anonymized to ensure confidentiality, as outlined in the study design. All steps of data extraction and analysis were documented. Hence, the procedures can be reproduced. Artificial intelligence–assisted tools, including Grammarly and ChatGPT, were used for language and grammar improvements.

### Definitions

COPD was diagnosed based on GOLD 2020^7^ guidelines, and patients were divided into statin users and nonusers. Statin users were defined as patients who used statins for at least 3 months at study inclusion and maintained 80% compliance during a 1-year follow-up.^8^ Adherence was evaluated using prescription records, and a compliance rate of 80% was verified.^9^ Statin nonusers comprised patients with COPD with no statin prescriptions between the index date and the end of the follow-up date. Acute exacerbation of COPD was defined by the need for additional therapy due to worsening symptoms, in accordance with GOLD guidelines and the treating physician’s judgment. Mild exacerbations were defined as those requiring a short-acting bronchodilator. Exacerbations requiring the use of antibiotics or oral steroids in addition to short-acting bronchodilators were classified as moderate. Severe exacerbations were defined as those requiring hospitalization or emergency department visits.^7^

### Outcome measures

The annual exacerbation rate and number of hospitalizations were considered primary outcomes. The number of outpatient visits and emergency visits due to COPD, the severity of exacerbation, and duration of inpatient stay due to COPD exacerbations were evaluated as secondary outcome parameters.

### Sample size

To achieve a clinically significant difference of 25% in exacerbation risk between the two arms (power: 80% and 95% confidence interval (CI)), 66 patients per arm were required. Eighty-three patients were recruited per arm to account for a 20% dropout, resulting in a total sample size of 166. Cases were allotted to the statin arm and the nonstatin arm in a 1:1 ratio if they met the inclusion criteria.

### Statistical analysis

**Figure 1.**
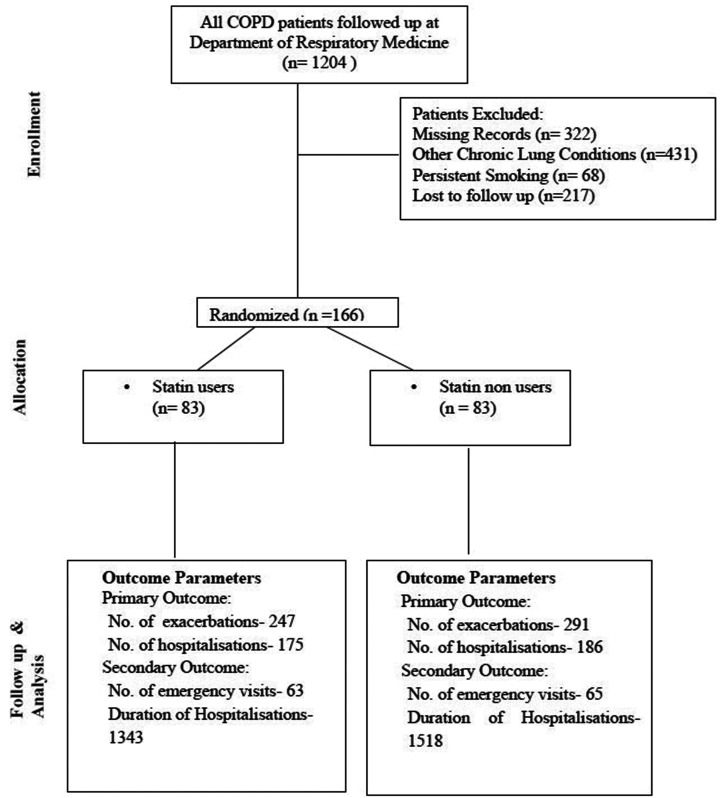
Consort flow diagram. Consort: Consolidated Standards of Reporting Trials.

The data were analyzed using Microsoft Excel. Data were presented as frequency (percentage) or as mean ± SD, in accordance with standard statistical practices. The chi-square test and the unpaired *t*-test were used to assess for statistical significance. The results were visually presented using graphs and tables, with the significance level set at p < 0.05. The reporting of this study conforms to the Strengthening the Reporting of Observational Studies in Epidemiology (STROBE) guidelines.^10^ The Consolidated Standards of Reporting Trials (CONSORT) flow diagram for the study is presented in Figure 1.

## Results

Patients with COPD were categorized into statin users and nonusers, with 83 patients in each group. The mean durations of follow-up among statin users versus nonusers were 15.93 ± 2.33 months and 15.29 ±2.43 months, respectively. Screening, enrollment, and follow-up of the study population are demonstrated in Figure 1. The baseline characteristics of the study population are presented in Table 1.

**Table 1.** Baseline characteristics of the study population.

	Statin users, N = 83	Statin nonusers, N = 83	p value
Age: years (median, range)	72 (67–78)	69 (64–75)	0.016
Sex, no. (%)			
Male	66 (79.5%)	70 (84.3%)	0.42
Female	17 (20.5%)	13 (15.7%)	
BMI, mean (kg/m^2^)	24.363	24.3555	0.99
Smoking status: no. (%)			
No data available	21 (25.3%)	26 (31.3%)	
Nonsmoker	41 (49.4%)	41 (49.4%)	0.495
Smoker	21 (25.3%)	16 (19.3%)	
Comorbidities: no. (%)			
Ischemic heart disease	65 (78.3%)	6 (7.2%)	0.001
Diabetes mellitus	39 (46.9%)	30 (36.14%)	0.88
Hypertension	6 (12%)	44 (53.0%)	0.001
Benign prostatic hypertrophy	6 (7.2%)	12 (14.4%)	0.05
Dyslipidemia	1 (1.2%)	0	0.99
Old cerebrovascular accidents	6 (7.2%)	3 (3.6%)	0.73
Obstructive sleep apnea	1 (1.2%)	3 (3.6%)	0.32
Hypothyroidism	4 (4.8%)	3 (3.6%)	0.99
Chronic kidney disease	4 (4.8%)	3 (3.6%)	0.99
COPD group: no. (%)			
A	1 (1.2%)	1 (1.2%)	>0.99
B	2 (2.4%)	1 (1.2%)	
C	2 (2.4%)	2 (2.4%)	
D	78 (94%)	79 (97.5%)	
GOLD grading: no.(%)			
I	7 (8.4%)	5 (6.0%)	
II	26 (31.3%)	23 (27.7%)	0.307
III	29 (34.9%)	21 (25.3%)	
IV	21 (25.3%)	34 (40.9 %)	
Post bronchodilator FEV1 predicted	0.52 ± 0.67	0.49 ± 0.63	0.869
Total duration of follow-up (months) mean	15.75	15.29	

A, B, C, and D—GOLD grouping of COPD. A (low risk, low symptoms), B (low risk, high symptoms), C (high risk, low symptoms), and D (high risk, high symptoms).

I, II, III, and IV—GOLD staging of COPD—GOLD 1 (Mild): FEV1 ≥80% predicted, GOLD II (Moderate): 50% ≤ FEV1 < 80% predicted, GOLD III (severe): 30% ≤ FEV1 < 50% predicted, GOLD IV (very severe): FEV1 < 30% predicted.

CI: confidence interval; COPD: chronic obstructive pulmonary disease; FEV1: forced expiratory volume in 1 s; GOLD: Global Initiative for Chronic Obstructive Lung Disease; SD: standard deviation.

**Figure 2.**
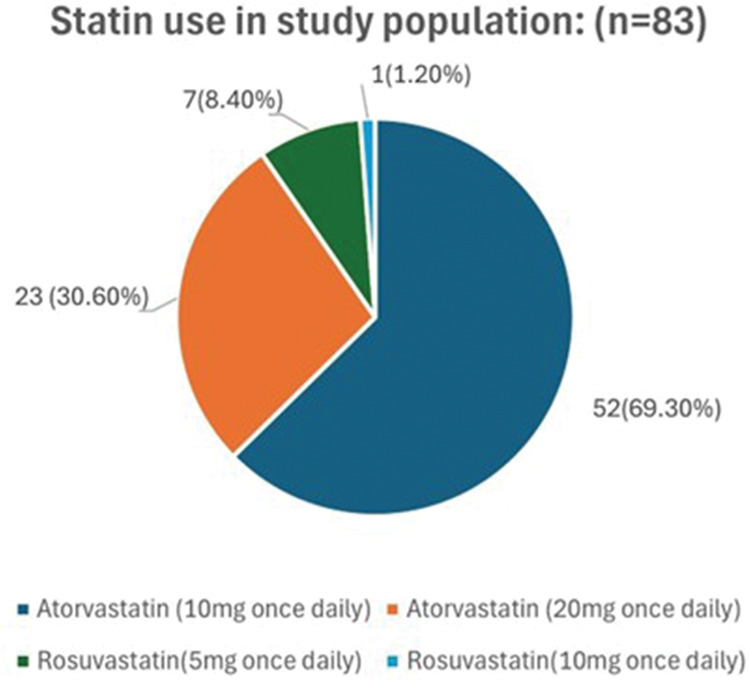
Statin use in the study population.

The most commonly prescribed statins included atorvastatin at a dose of 10–20 mg, followed by rosuvastatin (5–10 mg/day), as depicted in Figure 2. The median duration of statin therapy prior to inclusion was 11.2  (interquartile range: 9.8–13.4) months.

### COPD-related outcomes

**Figure 3.**
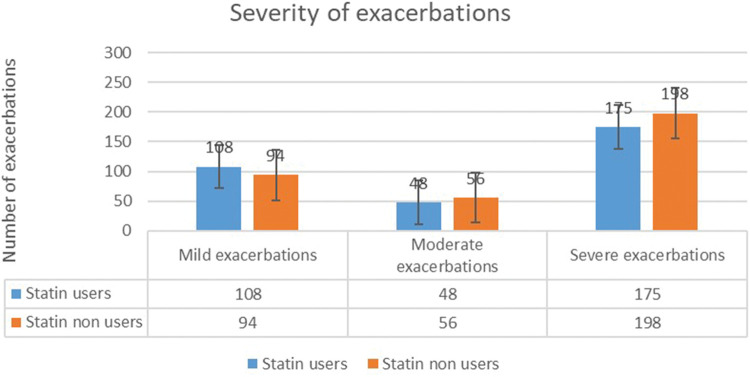
Severity of exacerbations among statin users and nonusers.

The annual exacerbation rate was marginally lower among statin users (2.98 ± 1.9) than among nonstatin users (3.51 ± 2.1), although this difference was not statistically significant (p = 0.058). As illustrated in Figure 3, statin users tended to have fewer moderate and severe exacerbations compared to nonusers. However, this difference was not statistically significant. The likelihood of exacerbations was 12% lower in patients using statins than in those not using statins (adjusted odds ratio: 0.88; 95% CI: 0.755–1.026). This association was, however, not statistically significant (p = 0.167).

As depicted in Table 2, the total inpatient stay for COPD exacerbations was shorter in statin users (1343 vs. 1518 days), which was statistically significant (p = 0.001). The numbers of outpatient visits (266 vs. 285, p = 0.418), hospitalizations (173 vs. 185, p = 0.493), and emergency room visits (63 vs. 65, p = 0.1) were slightly lower in statin users than in nonstatin users, although these differences were not statistically significant. The average hospital stay was lower in statin users (16.18 ± 13.7 days) than in nonstatin users (18.3 ± 16.05 days).

**Table 2.** COPD-related outcomes.

	Statin users (n = 83)	Nonusers(n = 83)		Statin users (n = 83)	Nonusers(n = 83)
Outcomes	Number(% age)	Number(% age)	p value^a^	Mean ± SD	Mean ± SD
Exacerbations at 1 year	247 (45.9%)	291 (54.1%)	0.058	2.98 ± 1.94	3.51 ± 2.18
Number of mild exacerbations	108 (53.5%)	94 (46.5%)	0.97	1.30 ± 2.01	1.13 ± 1.86
Number of moderate exacerbations	48 (46.1%)	56 (53.9%)	0.61	0.58 ± 1.140	0.67 ± 1.14
Number of severe exacerbations	75 (46.8%)	198 (53.2%)	0.21	2.11 ± 1.74	2.39 ± 1.92
COPD-specific OPD visits	236 (45.2%)	285 (54.8%)	0.418	3.20 ± 2.63	3.43 ± 2.48
COPD-specific hospitalizations	173 (48.4%)	186 (51.6%)	0.493	2.08 ± 1.62	2.24 ± 1.90
Duration of inpatient stay	1343 (46.9%)	1518 (53.1%)	0.001^a^	16.18 ± 13.78	18.29 ± 16.05
COPD-specific emergency visits	63 (49.2%)	65 (50.8%)	0.8	0.78 ± 1.19	0.76 ± 1.17

a p value calculated using chi-square test at 95% CI.

CI: confidence interval; COPD: chronic obstructive pulmonary disease; SD: standard deviation; OPD: outpatient department.

To assess the role of comorbidities as confounding factors, Poisson regression analysis was performed. Ischemic heart disease (IRR: 1.18; 95% CI: 0.91–1.53) and diabetes mellitus (IRR: 1.04, 95% CI: 0.86–1.25) did not show any significant association with exacerbations at 1 year after adjusting for statin use.

A subgroup analysis was conducted among patients with diabetes mellitus and COPD. The results of the subgroup analysis of patients with diabetes mellitus and COPD are depicted in Table 3. Although the annual exacerbation rate was slightly lower among statin users (3.08 ± 1.81 days) than among nonstatin users (3.34 ± 2.48 days), this difference was not statistically significant (p = 0.095). The duration of inpatient stay was also slightly shorter among statin users (17.92 ± 12.88 days) than among nonstatin users (20.66 ± 17.89 days), although this difference was not statistically significant (p = 0.747).

**Table 3.** Subgroup analysis of COPD-related outcomes in patients with diabetes on statin therapy.

	Statin users (n = 39)	Nonstatin users (n = 30)	
Outcomes	Mean ± SD	Mean ± SD	p value^a^
Annual exacerbation rate	3.08 ± 1.81	3.34 ± 2.48	0.0985
COPD-specific hospitalizations	2.26 ± 1.48	2.41 ± 2.18	0.678
Duration of inpatient stay	17.92 ± 12.88	20.66 ± 17.89	0.747

a p value calculated using the Mann–Whitney U test at 95% CI.

CI: confidence interval; COPD: chronic obstructive pulmonary disease; SD: standard deviation.

## Discussion

Statins suppress inflammatory and tissue-remodeling pathways by inhibiting guanosine triphosphatase and nuclear factor-kappa B (NFκB).^11^ They reduce cytokines (tumor necrosis factor α (TNF-α), IL-6, and IL-8) and neutrophil lung infiltration, limiting profibrotic activity that leads to small airway fibrosis and irreversible airflow obstruction in COPD.^12^ Additionally, IL-6–mediated antioxidant and anti-inflammatory effects may lower infection-related lung inflammation.^13,14^ Given these molecular-level mechanisms and their potential benefits, this retrospective cohort study was conducted to assess the effect of statins on COPD-related clinical outcomes in a subset of Indian population.

Similar to the RODEO trial, most participants in our study were elderly males, with a mean age of 70 years. Among statin users, the most common comorbidity was ischemic heart disease, whereas nonusers primarily had hypertension.^15^ The majority of our patients were classified as Group D under the GOLD classification for COPD, similar to the STATUETTE cohort study.^16^ The fact that the study was conducted at a tertiary care center, and hence, a referral bias is quite likely, and can explain the higher representation of Group D COPD patients. In addition, most of our COPD patients in our cohort belonged to GOLD grades III and IV. This contrasts with the COSYCONET cohort study,^17^ in which most participants were in Grades II and III. Hence, the results may not be directly applicable to patients with mild or moderate COPD or to the female population.

The literature regarding the effect of statins on COPD-related outcomes remains equivocal. In our study, there was a trend toward a reduction in the number and severity of acute exacerbations of COPD among statin users compared to nonusers, at the end of the 1-year follow-up period. However, this difference was not statistically significant. A study from Copenhagen, Denmark,^18^ and a meta-analysis by Horita et al.^19^ found that “statin use was associated with reduced odds of exacerbations.” However, in this study, statin use was not associated with reduced exacerbations in the most severe patients with COPD. The STATCOPE study demonstrated that “simvastatin did not affect exacerbation rates or the time to a first exacerbation in patients with COPD.”^20^ The much smaller RODEO trial,^15^ the COSYCONET cohort study,^17^ and a systematic review by Walsh et al.^21^ also failed to find any significant benefit of statin use on the rate of exacerbations.”

However, Ajmera et al.^22^ found a favorable impact on other COPD-related outcomes in adults receiving statins. They were less likely to have emergency visits, COPD-specific hospitalizations, and outpatient visits than those not receiving statins. Similarly, our study showed a significant reduction in duration of hospitalization and a lower (although not statistically significant) number of hospitalizations, outpatient visits, and emergency visits among statin users.

The COPD population is not homogeneous, and hence, it is possible that certain subsets may respond differently to statins. Cardiovascular disease is the leading cause of morbidity and mortality in patients with existing COPD and is associated with approximately 30%–50% of deaths in COPD patients.^23^ A study conducted on patients with COPD in Taiwan showed that” use of statins provided a protective effect against subsequent hospitalized exacerbations in male patients aged 75 years and older, with coexisting cardiovascular disease, but no protective effect was observed in those with no cardiovascular comorbidities.”^24^ These results point toward the fact that certain COPD subgroups may benefit from statin use, so far as COPD-related outcomes are concerned. The majority of statin users in our study population had significant cardiovascular comorbidities (p = 0.001*). However, using Poisson regression ischemic heart disease was not significant confounding factor for increased risk of exacerbations.

On subset analysis of diabetic patients with COPD, there was no significant difference in frequency and severity of exacerbations, number of outpatient or emergency visits, or duration of hospital stay between the two groups. However, in a Ukrainian cohort of patients with COPD with type 2 diabetes, adjunctive atorvastatin therapy significantly reduced the annual frequency of exacerbations as well as the number of emergency calls and hospital admissions.^25^ This difference may be due to the small sample size of patients with diabetes in our study. Furthermore, randomized controlled trials are required to assess the role of statins in patients with COPD who have other comorbidities such as cardiovascular disease and diabetes mellitus.

### Limitations

Study limitations include the possibility of missing data due to the retrospective study design and nonuniformity of data entry by different physicians. The findings are primarily representative of older male patients with severe COPD, thus limiting the generalizability to mild disease categories or the female population. The findings of this manuscript are based on a limited sample size and include a substantial number of patients with missing smoking history data, which may have contributed to the lack of statistically significant results. There was a biased selection of ischemic heart disease patients for the statin group in an otherwise comparable cohort since cardiovascular disease was the most common indication for statin prescription.

Statin adherence appeared to be 100% based on prescription records, although actual compliance is uncertain. Studies conducted by Ingebrigtsen TS and Lahousse^26^ use high CRP levels as a marker of systemic inflammation. These studies found that statin use was linked to lower odds of having a high CRP, and a high CRP was associated with a higher risk of exacerbations. However, these inflammatory markers were not assessed in our study, due to nonavailability of data. Additionally, as the data have been collected from a single center, referral bias and limited external validity should be considered.

## Conclusion

There was a trend, although not statistically significant, toward reduced exacerbations at the end of 1-year follow-up among statin users. The total duration of inpatient stay was also significantly shorter. Reduced number of outpatient visits and severe exacerbations were seen in statin users, but again, not statistically significant.

### Main points

- Statins with their anti-inflammatory and immunomodulatory properties may be of value in the management of COPD.
- This study aims to analyze the effects of statins on COPD-related outcomes such as exacerbations and hospitalizations in a section of Indian population.
- An overall trend toward a reduced number of exacerbations at the end of the 1-year follow-up period was observed among the statin users, although not statistically significant.
- The total duration of inpatient stay was also shorter among the statin users.

## Data Availability

The data supporting the findings of this study are available from the corresponding author upon reasonable request.
